# The role of serum ADAMTS-1 levels in Hyperemesis Gravidarum

**DOI:** 10.1186/s12884-022-04832-7

**Published:** 2022-06-20

**Authors:** Burcu Timur, Gurhan Guney

**Affiliations:** 1 Department of Obstetrics and Gynecology, Ordu University Training and Research Hospital, Bucak District, Nefsi Bucak Street, Ordu, 52200 Turkey; 2 Department of Obstetrics and Gynecology, Balikesir University Medical Faculty, Balıkesir, Turkey

**Keywords:** Hyperemesis gravidarum, ADAMTS-1 protein, Ketosis, Pregnancy, Placenta

## Abstract

**Background:**

We aimed to investigate the levels of ADAMTS-1, which is secreted from the extracellular matrix during trophoblastic invasion in hyperemesis gravidarum (HEG).

**Methods:**

In this cross-sectional study, we compared 45 HEG patients aged between 21 and 34 in terms of ADAMTS-1 levels with a control group consisting of 44 healthy pregnant women. The demographic characteristics and several laboratory parameters of the patients were recorded. Both groups were also compared in terms of ketonuria. We evaluated the correlation between ADAMTS-1 levels and ketonuria.

**Results:**

The 2 groups were matched in terms of age, gestational age, gravidity, parity, and body mass index. Some inflammatory markers, such as neutrophil count, MPV, PDW, and PCT levels, were significantly higher in the HEG groups compared to the control group (all *p* < 0.05). However, mean MCV and serum TSH levels were statistically significantly lower in this group (both *p* < 0.001). ADAMTS-1 levels were 12.6 ± 1.4 ng/ml in the HEG group and 6.2 ± 1.6 ng/ml in the control group (*p* < 0.001). It was significantly and positively correlated with urine ketone, neutrophil count, and PDW, whereas negatively correlated with MCV and TSH value in the HEG group. ROC analysis showed that a threshold value of 11.275 ng/ml for ADAMTS-1 predicted HEG patients with a sensitivity of 60% and specificity of 95.5%.

**Conclusion:**

ADAMTS-1 serum levels are increased in HEG patients, and there is a positive correlation between ADAMTS-1 levels and ketonuria.

## Background

Hyperemesis gravidarum (HEG), representing the severe end of the spectrum associated with nausea and vomiting in pregnancy, refers to persistent vomiting resulting in ketonuria and/or ketonemia leading to weight loss and volume reduction exceeding 5% of pre-pregnancy weight [1]. HEG, which occurs in about two percent of all pregnancies in the United States, may cause the pregnant woman to be unable to do her daily work, lead to anxiety and depression, compel some patients to terminate the pregnancy, and avoid planning future pregnancies [2]. In addition to its negative effects on the mother, it is also associated with poor fetal outcomes, such as preterm birth, neuronal developmental delay, and embryopathy due to vitamin K deficiency [3]. HEG is the most common reason for hospitalization in the first trimester of pregnancy, and it has an economic burden on the health system [4, 5].

HEG is not observed in every patient with high hCG values. In the studies, it was stated that different isoforms and/or different receptors of hCG and their interactions with other molecules secreted in the extracellular environment may be responsible for the difference in hyperemesis symptoms, but no clear result has been revealed. Importantly, in the serum and genetic studies, hCG was also studied and was not associated with HEG. If different isoforms were involved, these isoforms should have shown up in the genetic studies, but they did not [6].

Recently the nausea and vomiting hormone GDF15 has been proven to be the most likely causal factor for HEG and hCG has been much ruled out. GDF15 is expressed by trophoblasts, increases during placentation, is increased in maternal serum in HEG patients and patients with more severe and/or prolonged vomiting, and has now been associated with HEG in 2 genetic studies [7, 8]. Intermediate trophoblasts, which are formed by the transformation of cytotrophoblasts, initiate both extracellular and endovascular invasion on the feto-maternal face and secrete GDF15 into maternal blood. During this invasion, proteolytic destruction of the extracellular matrix is seen, and various numbers of matrix metalloproteinases (MMP) play roles in this placentation process. One of the proteinases that regulates the activity of MMP is the A Disintegrin and Metalloproteinase with Thrombospondin Motifs (ADAMTS) family. ADAMTS-1, the first member of this group, modulates angiogenesis by binding to vascular endothelial growth factor (VEGF), and plays a role in both pregnancy formation and the continuation of a healthy pregnancy by adjusting the depth of trophoblastic invasion. In studies, ADAMTS-1 was found to be associated with the terminal differentiation of cytotrophoblasts and their transformation into an invasive phenotype as observed in gestational trophoblastic diseases [9]. ADAMTS-1 showed strong immunohistochemical staining on the maternal fetal face, but in unhealthy pregnancies, such as miscarriages and anembryonic pregnancies, it was weakly stained. So ADAMTS-1 is needed for a pregnancy to approach full-term [9, 10].

ADAMTS-1 was originally identified in a cachexia model and cachexia has overlapping symptoms with HEG (appetite loss, weight loss, and muscle wasting) [11]. Therefore, we aimed to confirm whether if ADAMTS-1 has a role in the development of HEG. Moreover, the relationship between ADAMTS-1 and other biochemical parameters known to be associated with HEG were investigated.

## Methods

This study was approved by the Gumushane University Ethics Committee(Project number:2022/E-95674917) and was conducted in accordance with the Declaration of Helsinki. All participants included in the study gave written and verbal informed consent prior to being enrolled in the study.

### Patients

In this cross-sectional study, the HEG group consisted of 45 patients aged between 21 and 34 who were admitted for nause and vomiting during pregnancy to the Ordu University Education and Research Hospital Gynecology and Obstetrics Clinic between January 2018 and January 2022. The control group consisted of 44 pregnant women who applied to the same clinic for routine antenatal follow up and were matched in terms of chronological age, gestational age, and body mass index (BMI). Patients with diabetes or obesity, peptic ulcer or gastritis, liver and/or gallbladder disease (cholangitis or gallstones), thyroid dysfunction, urinary tract infection, coeliac disease, cardiovascular or renal diseases were excluded from our study. We also excluded any patients who has nausea and vomiting and any patients who has threatened miscarriage,as vaginal bleeding during pregnancy may affect ADAMTS levels [12]. Multiple pregnancies and/or pregnancies obtained by assisted reproductive techniques, gestational trophoblastic disease, and autoimmune disease conditions were also not included in the study.

HEG was diagnosed if there was a persistent vomiting resulting in ketonuria and/or ketonemia leading to weight loss and volume reduction exceeding 5% of pre-pregnancy weight.

Women who vomited more than 3 times a day due to HEG and/or who had a positive urine ketone test result and/or were hospitalized for HEG 2 or more times. BMI was measured by dividing the weight in kilograms by the height in meters squared (kg/m^2^). Doctors performed the first transabdominal pregnancy ultrasonography using a 3.5 MHz transabdominal transducer (General Electric Logiq A5 ultrasound machine, Milwaukee, USA), and the gestational age was estimated according to the first trimester ultrasonographic value of fetal crown-rump length.

### Laboratory studies

All laboratory analyses for each patient were performed in the early morning after an overnight fast of 8–12 h. We first hydrated the patients with 1000 cc RL solution and collected serum samples after sufficient urine output. We did not dilute the serum samples.

We evaluated complete blood count, thyroid-stimulating hormone (TSH), T3, T4, fasting plasma glucose, BMI, aspartate transferase, alanine transferase (ALT), blood urea nitrogen (BUN), and creatinine levels.

### Measurement of serum ADAMTS-1 Levels

Venous blood samples were collected from each participant’s antecubital vein after at least 8 h of fasting and then separated by centrifugation at 4000 rpm for 10 min. In order to measure serum ADAMTS-1 protease, we obtained blood samples after we diagnosed HEG and then we stored the materials at -80 ˚C until the study time. Frozen serum samples were thawed to room temperature and were not diluted. ADAMTS-1 levels were measured with a commercially available enzyme-linked immunosorbent assay (ELISA) kit (Hangzhou Eastbiopharm Co., Ltd., China) and this ELISA kit was specific for ADAMTS-1.The test procedure was completed in accordance with the manufacturer’s instructions using biotin double-antibody sandwich technology. Serum samples were added onto wells that were previously coated with ADAMTS-1 monoclonal antibodies. Biotin labelled anti-ADAMTS-1 antibodies and streptavidin-HRP were added to form immune complexes. Following incubation, unbound enzymes were removed by washing. Chromogen solutions A and B were then added. Stop solution was used to stop the reaction and formed yellow color’s optical density was measured by using a microplate reader at 450 nm, which was proportional to the amount of ADAMTS-1. ADAMTS-1 serum levels, as ng/ml, were interpolated from a standard curve. The intra-and interassay variation coefficients of the kit were smaller than 9% and 11%, respectively.

### Statistical analyses

Statistical analysis was performed using SPSS version 22. Descriptive statistics, such as mean, standard deviation, and median (minimum–maximum), were used while statistically defining normal and non-normal variables. Normality was tested using the Shapiro–Wilk test. After performing the normality test, parametric (independent samples t-test) and non-parametric (Mann–Whitney U) analysis tests were used to detect the differences between the groups. Spearman’s rank-order correlation method was used for univariate correlations. To reveal the predictive value of ADAMTS-1 protein for HEG, the receiver operator characteristics curve (ROC) analysis was applied. A priori power analysis for the two independent means was done using G*Power software (Universitat Kiel, Kiel, Germany) to define an adequate sample size with a large effect size (α: 0.05 and β: 0.95). We considered *p* value less than 0.05 as statistically significant.

## Results

In our current cross-sectional study, we compared 45 HEG patients with a control group consisting of 44 healthy pregnant women without nausea and vomiting caused by pregnancy. Table 1 shows the demographics and clinical characteristics of both groups. There was no difference between the two groups in terms of age (26.5 ± 5 vs. 28.5 ± 6, p:0.084), gestational week (8.1 ± 4.8 vs. 9.0 ± 5.1 weeks, p:0.904), gravida (2 ± 1.5 vs. 2.1 ± 2, p:0.505), and BMI (24.0 ± 3.8 vs. 25.3 ± 3.7, p:0.086). Table 2 shows the biochemical characteristics of both groups. There was an insignificant difference between the two groups in terms of hemoglobin, hematocrit, platelet, AST, ALT, BUN, creatinine, and fasting glucose values (all *p* > 0.05). When compared with the control group, MPV (10.0 ± 1.4 fl vs. 8.8 ± 1.0 fl), PCT (0.26 ± 0.06 vs. 0.22 ± 0.05), PDW (16.0 ± 0.5 vs. 14.4 ± 0.4), and free T4 (1.2 ± 0.5 ng/dl vs. 1.0 ± 0.2 ng/dl) values were significantly higher (*p* < 0.001, p:0.003, p:0.002, and p:0.001, respectively), while the MCV (82.7 ± 5.7 fl vs. 85.6 ± 5.9 fl) and TSH (0.9 ± 0.7 mU/l vs. 1.6 ± 1.2 mU/l) values were significantly lower in HEG group (p:0.020 and p:0.001, respectively). Urine ketone levels were significantly higher in HEG group than in the control group. We also observed that ADAMTS-1 levels were significantly higher in the HEG group compared to the control group (12.6 ± 3.6 ng/dl vs. 6.2 ± 1.4 ng/dl, *p* < 0.001).

**Table 1:** Demographics and clinical characteristics of the groups

Variable	HEG (n:45)	CONTROL (n::44)	P
Age(years)	26.5 ± 5	28.5 ± 6	0.084
Gravida	2 ± 1.5	2 ± 2	0.515
Parity	0 ± 1	1 ± 1	0.188
No of Living Child	0 ± 1	1 ± 1	0.150
No of Abortion	0 ± 1	0 ± 0	0.373
Gestational weeks	8.1 ± 4.8	9.0 ± 5.1	0.904
BMI (kg/m^2^)	23.9 ± 3.8	25.3 ± 3.7	0.086

Data presented as Mean ± SD

*P* < 0.05 accepted as significant

**Table 2:** Laboratory parameters of the groups

Variable	HEG (n:45)	CONTROL (n::44)	P
Urine ketone	80 ± 55(40–150)	0	** < 0.001**
Blood glucose	96.4 ± 26.5	90.3 ± 10.0	0.154
BUN	21.4 ± 6.7	19.0 ± 5.6	0.069
Creatinine	0.6 ± 0.1	0.6 ± 0.1	0.693
AST	24.9 ± 29.0	20.6 ± 4.8	0.342
ALT	26.5 ± 37.6	16.1 ± 11.1	0.086
WBC	8.6 ± 2.3	8.9 ± 1.7	0.576
Neutrophile	74.4 ± 7.5	68.1 ± 9.2	**0.001**
Lymphocyte	23.1 ± 2.63	22.5 ± 5.7	0.915
Monocyte	5.3 ± 1.8	7.1 ± 6.6	0.093
Hb	13.3 ± 1.1	13.1 ± 1.2	0.211
Hct	38.7 ± 3.0	38.6 ± 2.8	0.882
MCV	82.7 ± 5.7	85.6 ± 5.9	**0.020**
RDW	14.0 ± 2.7	14.5 ± 3.2	0.417
PLT	268.2 ± 72.3	257.1 ± 62.3	0.437
MPV	10.0 ± 1.4	8.8 ± 1.0	** < 0.001**
PCT	0.26 ± 0.06	0.22 ± 0.05	**0.003**
PDW	16.0 ± 0.5	14.4 ± 0.4	**0.002**
Free T3	3.3 ± 0.9	3.1 ± 0.3	0.135
Free T4	1.2 ± 0.5	1.0 ± 0.2	**0.001**
TSH	0.9 ± 0.7	1.6 ± 1.2	**0.001**
ADAMTS-1	12.6 ± 3.6	6.2 ± 1.4	** < 0.001**

Spearman’s rank-order correlation also showed that ADAMTS-1 levels were significantly and positively correlated with urine ketone, neutrophil count, PDW, and free T4 levels, whereas they were negatively correlated with MCV and TSH values in the HEG group (Table 3).

**Table 3:** Correlation analysis

	**Ketone**	**Neutrophile**	**MCV**	**PCT**	**Free T4**	**TSH**
ADAMTS-1						
r	0.371	0.229	-0.248	0.336	0.302	-0.314
p	< 0.001	0.031	0.019	0.001	0.004	0.003

*P* < 0.05 accepted as significant, *r* Correlation coefficient

**Fig. 1:**
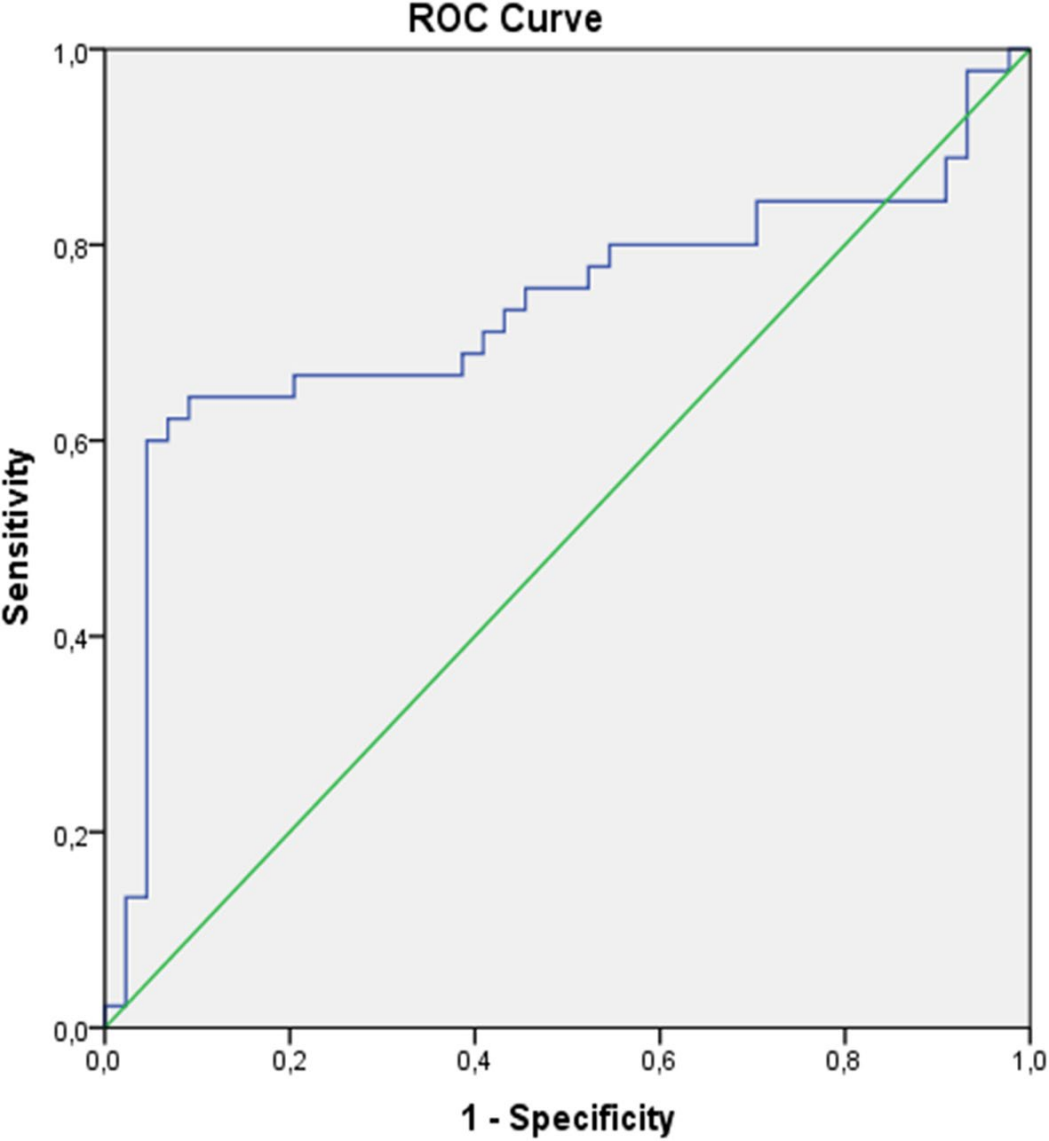
ROC curve analysis of ADAMTS-1 levels

The ROC analysis showed that the area under the ROC curve (AUC) was 0.731 (0.619–0.843, 95% confidence interval). This means that ADAMTS-1 is a useful biomarker for distinguishing pregnant women diagnosed with HEG (*p* < 0.001). The cut-off value according to the highest Youden’s index was calculated to be 11.275 ng/ml for ADAMTS-1 with a sensitivity of 60% and specificity of 95.5% (Fig. 1).

## Discussion

In this study, we showed that serum ADAMTS-1 levels were significantly increased in HEG patients compared with the healthy pregnant control group. There was a significant and positive correlation with urine ketone levels and a significant negative correlation with serum TSH levels. Our study is the first to evaluate ADAMTS-1 levels in HEG patients and their associations with other biochemical parameters.

Liu et al. [13] stated in their study that not only hCG but also many different cytokines are secreted from trophoblasts in chorionic villi, and hCG alone may not be responsible for nausea and vomiting. For example, in a study by Fejzo M.S et al. [14] it was found that the GDF-15 gene encodes a TGF-β superfamily member, which is expressed at high levels in the trophoblast cells of the placenta, and this protein, which has high levels in maternal blood especially in the first two trimesters, acts to facilitate placentation and suppress pro-inflammatory production of cytokines in order to suppress the continuation of pregnancy. In addition to this role, in the same study, it was shown that GDF 15 activates hypothalamic neurons and regulates body weight and appetite by affecting the nausea-vomiting center in the area postrema. In a study conducted by Kuno et al. [11] and Lerner L et al. [15], which included the same model of cachexia, they showed that overproduction of GDF-15 in cancer patients was the main cause of cancer anorexia and cachexia, and it led to the symptoms of chronic nausea and vomiting in HEG.They also found that ADAMTS-1 and GDF-15 together may be associated with cancer cachexia. Thus, in the future it will be interesting to determine whether ADAMTS-1 increases GDF15 levels or vise versa. It has been shown in various studies that the presence of ADAMTS-1 is needed for a healthy implantation, placentation, and continuation of pregnancy [10, 16, 17]. In a large population-based cohort study, it was reported that the incidence of small-for-gestational age (SGA) increased in women with HEG [18]. It has been claimed that this may be secondary to placentation abnormality and stated that this possibility is stronger in the presence of HEG and early-onset preeclampsia, especially in the second trimester [18]. In the literature, there is no study investigating the relationship between ADAMTS-1 levels and HEG, but there was a study investigating its levels in preeclampsia patients. In this study by Kalem et al. [9], the ADAMTS-1 levels in the blood of preeclamptic patients were lower, and the levels in the placental tissue were higher than in the control group. In our study, we found ADAMTS-1 levels to be significantly higher in HEG patients than in the control group. Based on this, crosstalk between trophoblasts and the extracellular matrix might be correlated with ADAMTS-1 levels rather than trophoblast invasion in HEG. It is well known that ADAMTS-1 functions as a VEGF antagonist [19]. VEGF, on the other hand, is a vascular permeability factor that plays a role in angiogenesis, which is produced in the maternal fetal unit at high levels and secreted into the maternal blood during pregnancy. Schreurs et al. [20] showed that VEGF had the feature of increasing the blood–brain barrier. In a different study, Miyata et al. [21] showed that the blood–brain barrier, which is more permeable than other parts of the brain as formed by fenestrated capillaries, could become more permeable with VEGF, especially in circumventricular organs such as the area postrema, which includes the nausea and vomiting center inside. Despite the relationship mentioned above, we could not find any study investigating VEGF levels in patients with HEG. Therefore, based on the above studies, we thought that ADAMTS-1 levels, which are high in HEG patients, might be secreted from the placenta as a compensation mechanism to regulate the increased permeability in the nausea-vomiting center. In a small number of studies on ADAMTS-1, it has been shown that by regulating the blood–brain barrier, it enables neurons to adapt to the new situation [22]. Although higher levels of ADAMTS-1 as an VEGF antagonist, would hypothetically result in decreased permeability which should decrease nausea and vomiting, ADAMTS-1 antagonizes VEGF to regulate trophoblastic invasion at the receptor level in the placenta. Considering that the VEGF receptor types in placental and neural tissues are different, it is possible that ADAMTS-1 may not be effective on VEGF receptors in the brain, or GDF-15 or some other growth factors and or cytokines may reduce the activity of ADAMTS-1 [10, 23].

It is well known that ketone bodies are a fuel for the brain as an alternative to glucose in long periods of fasting and have the ability to cross the blood–brain barrier [24]. Studies have shown that starvation ketosis could be observed in long-term nausea and vomiting seen in HEG, and this might be harmful to the maternal and fetal neuronal system [25]. ADAMTS-1 might have a function, such as protecting the blood–brain barrier from the negative effects of ketone bodies, because ADAMTS-1 is an antagonist of VEGF, which increases the blood–brain barrier. Although most studies could not find a relationship between ketonuria and HEG severity, we detected a significant degree of ketonuria in our HEG group compared to the control group, and we observed a positive correlation between ketonuria and ADAMTS-1 levels [26]. In a different study that investigated the relation between ketonuria and HEG, Soysal et al. [27] claimed that ketonuria in HEG was determined by the inflammatory response in pregnancy rather than HEG itself. Similar to that study, we found that inflammatory markers such as PDW, PCT, and MPV were higher in the HEG group compared with the control group.

One of the main weaknesses of this study is that GDF 15 levels were not measured. Another weakness is that we did not measure VEGF levels as there was no standardized value in the first trimester of pregnancy. The well-matched features of the patients and the fact that they were free from the effects of BMI and age showed that the results and comparisons were reliable.

## Conclusions

This study was the first clinical study to measure ADAMTS-1 levels in HEG patients. ADAMTS-1 levels were higher in HEG patients and positively correlated with ketonuria in this group of patients. Further comprehensive studies including the relationship of ADAMTS-1 with more patients in HEG and its relation with GDF-15 are recommended to reveal a clearer understanding of this subject.

## Data Availability

The datasets used and/or analysed during the current study are available from the corresponding author on reasonable request.

## Abbreviations

ADAMTS-1: A disintegrin and metalloproteinase with thrombospondin motifs 1
HEG: Hyperemesis gravidarum
MPV: Mean platelet volume
PDW: Platelet distribution width
PCT: Plateletcrit
MMP: Matrix metalloproteinases
ROC: Receiver operating characteristic
BMI: Body mass index
TSH: Thyroid stimulating hormone
